# Antioxidant Activity and Phytochemical Composition of the Leaves of *Solanum guaraniticum* A. St.-Hil

**DOI:** 10.3390/molecules171112560

**Published:** 2012-10-24

**Authors:** Marina Zadra, Mariana Piana, Thiele Faccim de Brum, Aline Augusti Boligon, Robson Borba de Freitas, Michel Mansur Machado, Sílvio Terra Stefanello, Félix Alexandre Antunes Soares, Margareth Linde Athayde

**Affiliations:** 1 Post-Graduate Program in Pharmaceutical Sciences, Federal University of Santa Maria, Camobi Campus, Santa Maria, RS, 97105-900, Brazil; 2 Post-Graduate Program in Pharmaceutical Sciences, Federal University of Pampa-UNIPAMPA, Uruguaiana, RS 97500-970, Brazil; 3 Post-Graduate Program in Biochemical Toxicology, Federal University of Santa Maria, Camobi Campus, Santa Maria, RS, 97105-900, Brazil

**Keywords:** *Solanum guaraniticum*, antioxidant activity, polyphenols, HPLC/DAD

## Abstract

*Solanum guaraniticum* is a shrub belonging to the Solanaceae family popularly known in Brazil as jurubeba or false-jurubeba. The aim of this study was to evaluate the antioxidant activity of crude extract and chloroform, ethyl acetate and *n*-butanol fractions from its leaves, verifying the ability to remove reactive species and identify and quantify phenolic compounds. The ethyl acetate fraction showed the highest amount of total polyphenols (546.57 ± 2.35 mg gallic acid equivalent/g) and the lowest IC_50_ (9.11 ± 0.75 µg/mL) by the DPPH method. Furthermore, the chloroform fraction presented the highest content of flavonoids (75.73 ± 0.34 mg rutin equivalents/g), tannins (56.03 ± 0.68 mg catechin equivalents/g) and alkaloids (10.79 ± 0.06 mg/g). This fraction was effective in the scavenging of reactive species by 2′,7′-dichlorofluorescein diacetate assay, in addition to completely reducing protein carbonyl content and reducing lipid peroxidation at basal levels even at low concentrations. Chlorogenic, caffeic and rosmarinic acids were identified and quantified by HPLC/DAD. These results show that *S. guaraniticum* is rich in phenolic compounds and has potential as an antioxidant.

## 1. Introduction

Medicinal plants have been widely used for therapeutic purposes since ancient times. The beneficial effects of fruits and vegetables are generally attributed to the presence of phenolic compounds such as phenolic acids, flavonoids and tannins, nitrogen compounds such as alkaloids and amines, as well as vitamins, terpenoids and other metabolites, which have a high antioxidant activity [1,2,3]. This activity is due to the ability of these substances to reduce oxidative stress by neutralizing or scavenging of reactive species by hydrogen donation, before they attack cells and other biological components [4].

Reactive oxygen (ROS) and nitrogen (RNS) species are products of normal cellular metabolism. However, at high concentrations, these species may be important mediators of damage to cellular structures, such as nucleic acids, lipids and proteins. The oxidation of any of these substrates, if uncontrolled, can contribute to the development of chronic diseases such as cancer, hypertension, diabetes mellitus, cardiovascular and neurodegenerative diseases [5,6]. In this sense, there is great interest in finding natural antioxidants from plant materials, and various extracts and isolated compounds have been investigated for their antioxidant activity, using different methods [7,8,9,10].

The Solanaceae family is one of the largest and most complex of the Angiosperms, and its main center of diversity and endemism is South America. It has species rich in active secondary metabolites and very important from economic, agricultural, and pharmaceutical point of view, especially the *Solanum* genus, one of the largest in the plant kingdom. Plants of this genus exhibit a wide variety of steroidal saponins and glycoalkaloids, furthermore, flavonoids are also frequently found. Due its different biological activities, its species have been extensively studied [11,12].

*Solanum guaraniticum* A. St.-Hil. (syn. *Solanum fastigiatum* var. *acicularium* Dunal) is a shrub popularly known as jurubeba or false-jurubeba, which occurs in Paraguay, Argentina and Brazil, and can be found on roadsides, forests and clear fields [13]. In popular medicine, its leaves, roots and fruits are used as tea in the treatment of anemia, fevers, erysipela, spleen and liver diseases such as hepatitis, ulcers, and uterine tumors, as tonic and as a digestive stimulant [14,15]. A previous study demonstrated that the infusion of the leaves has hepatoprotective and antioxidant activity *in vivo*, observed in male albino mice [16]. The Brazilian Pharmacopoeia only recognizes *Solanum paniculatum* as the true jurubeba, although in Brazil the general population uses both species as folklore medicines to treat liver diseases. Therefore, considering the interchangeable use of these two species and the scarce data available about *S. guaraniticum*, a deep evaluation of the antioxidant activity by different methods is required for a better understanding of its biological potential activities. Thus, the aim of this study was to quantify total polyphenols, flavonoids, tannins and alkaloids in the crude extract (CE) and chloroform (CHCl_3_), ethyl acetate (AcOEt) and *n*-butanol (n-BuOH) fractions from the leaves of *Solanum guaraniticum*, and to evaluate the antioxidant activity by the 1,1-diphenyl-2-picrylhydrazyl (DPPH), inhibition of lipid peroxidation (TBARS) and protein oxidation (carbonyl groups) assays. In addition, the ROS scavenging capacity was evaluated by using the 2′,7′-dichlorofluorescein diacetate (DCFH-DA) oxidation test. Taking into account phytochemical analysis, polyphenols in the different fractions were identified and quantified by high performance liquid chromatography-diode array detector (HPLC/DAD).

## 2. Results and Discussion

### 2.1. Phytochemical Composition

The contents of total polyphenols, flavonoids, condensed tannins and alkaloids are shown in Table 1. The AcOEt fraction presented the highest content of total polyphenols (546.57 ± 2.35 mg/g gallic acid equivalents (GAE)). The chloroform fraction, despite presenting the lowest content of total polyphenols, demonstrated the highest content of flavonoids, tannins and alkaloids. Similar findings were related by Janovik *et al.* [17], analyzing different fractions of *Cariniana domestica*. Alkaloids were only found in the CE and CHCl_3_ fractions.

**Table 1:** Total polyphenols (TP), total flavonoids (TF), condensed tannins (CT) and total alkaloids (TA) in the CE and fractions from leaves of *S. guaraniticum.*

Extract or Fraction	TP (mg GAE/g)	TF (mg RE/g)	CT (mg CaE/g)	TA (mg/g)
CE	259.95 ^b^ ± 0.69	61.30 ^b^ ± 0.53	23.16 ^b^ ± 2.05	6.14 ^b^ ± 0.01
CHCl_3_	195.90 ^c^ ± 1.24	75.73 ^a^ ± 0.34	56.03 ^a^ ± 0.68	10.79 ^a^ ± 0.06
AcOEt	546.57 ^a^ ± 2.35	57.17 ^c^ ± 0.07	11.85 ^c^ ± 0.91	-
*n*-BuOH	259.82 ^b^ ± 2.17	60.17 ^b^ ± 0.32	8.85 ^c^ ± 1.01	-

Values are expressed as mean ± standard deviation. GAE: gallic acid equivalents, RE: rutin equivalents, CaE: catechin equivalents, ^a–c^ Means with the different letters in each column are significantly different (*p* < 0.05), by analysis of variance (One-way ANOVA) (n = 3).

### 2.2. Radical Scavenging Capacity-DPPH Assay

**Figure 1:**
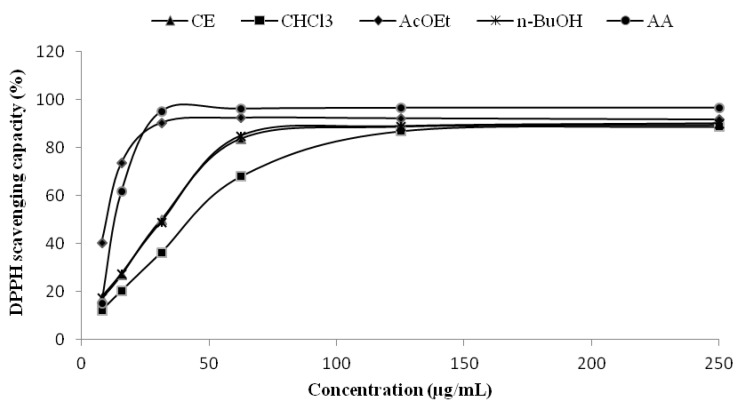
Antioxidant capacity of CE and fractions from leaves of* S. guaraniticum* by DPPH assay(n = 3).

The DPPH assay is based on the measurement of the ability of an antioxidant substance to scavenge the radical, reducing it to hydrazine, with a simultaneous change in color from violet to yellow, which is measured spectrophotometrically. The AcOEt fraction showed excellent antioxidant capacity, superior even to the standard ascorbic acid, with an IC_50_ of 9.11 ± 0.75 µg/mL while the standard had an IC_50_ of 15.48 ± 1.28 µg/mL (Figure 1 and Table 2). The CE and *n*-BuOH fractions showed very similar IC_50_ values, and finally, with higher IC_50_ but still a relatively low value, came the CHCl_3_ fraction (44.46 ± 1.27 µg/mL). These results are in agreement with the content of polyphenols found in the fractions (AcOEt > CE ≈ *n*-BuOH > CHCl_3_), so is possible to attribute the antioxidant capacity seen in the DPPH assay to the presence of these compounds. This relationship is already well established and has been described by other authors, using similar assay systems [18,19].

### 2.3. Scavenging of ROS-DCFH-DA Method

Dichlorofluorescein (DCFH) is widely used to determine oxidative stress in cells. When its diacetate form (DCFH-DA) is added to the cells, it diffuses through the cell membrane and is hydrolyzed by intracellular esterase to liberate DCFH, which reacts with oxidizing species (particularly H_2_O_2_ and hydroxyl radical) forming the fluorescent compound 2′,7′-dichlorofluorescein (DCF) [20,21]. Thus, the principle of this assay is to evaluate the ability of antioxidant molecules in the extract to scavenge ROS produced by normal metabolism by cells, and then inhibit the oxidation of DCFH to DCF, observed by the decrease in fluorescence intensity.

**Table 2:** IC_50_ values for DPPH (1), TBARS (2) and Carbonyl (3) assays.

Extract or Fraction	IC_50_^1^ (µg/mL)	IC_50_^2^ (µg/mL)	IC_50_^3^ (µg/mL)
CE	31.43 ^c^ ± 1.02	54.23 ^c^ ± 1.58	82.98 ^b^ ± 0.31
CHCl_3_	44.46 ^d^ ± 1.27	3.85 ^a^ ± 0.93	67.65 ^a^ ± 0.82
AcOEt	9.11 ^a^ ± 0.75	12.24 ^b^± 1.76	179.59 ^c^ ± 1.14
n-BuOH	32.12 ^c^ ± 0.91	55.10 ^c^ ± 1.94	87.22 ^b^ ± 0.32
AA	15.48 ^b^ ±1.28	117.81 ^d^ ± 1.23	61.80 ^a^ ± 0.54

AA: ascorbic acid. ^a^^–d^ Means with the different letters in each column are significantly different (*p* < 0.05), by analysis of variance (One-way ANOVA) (n = 3).

**Figure 2:**
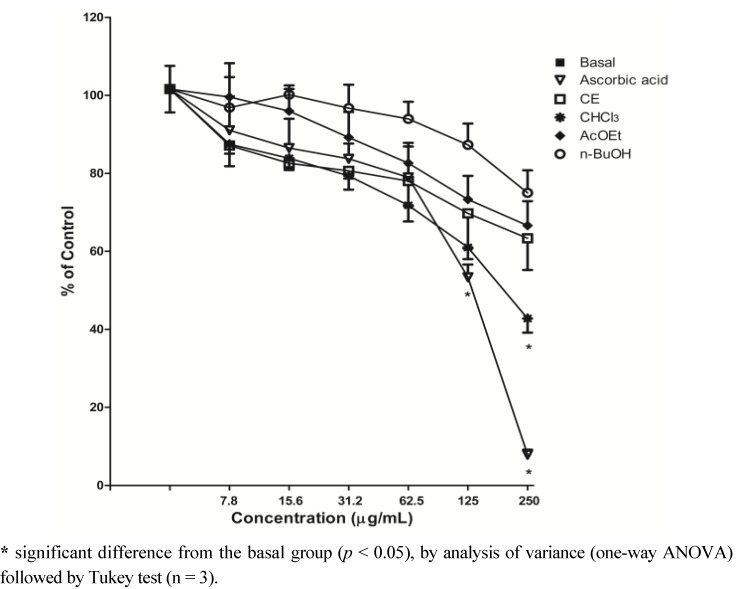
Effect of *S. guaraniticum* CE and fractions on scavenging of ROS in supernatant of rat brain homogenate, by DCFH-DA method.

The CHCl_3_ fraction at a concentration of 250 µg/mL was able to significantly reduce the oxidation of DCFH and consequently reduce the oxidative stress observed in supernatant of rat brain homogenate, compared to the basal group (*p* < 0.05), demonstrating pronounced antioxidant activity. Ascorbic acid reduced in a significant manner the oxidation of DCFH-DA, at 125 and 250 μg/mL (*p* < 0.05). The CE and other fractions did not demonstrate this ability; no statistically significant difference compared to the basal group at the same concentrations tested was observed (Figure 2).

### 2.4. Inhibition of Lipid Peroxidation-TBARS Assay

**Figure 3:**
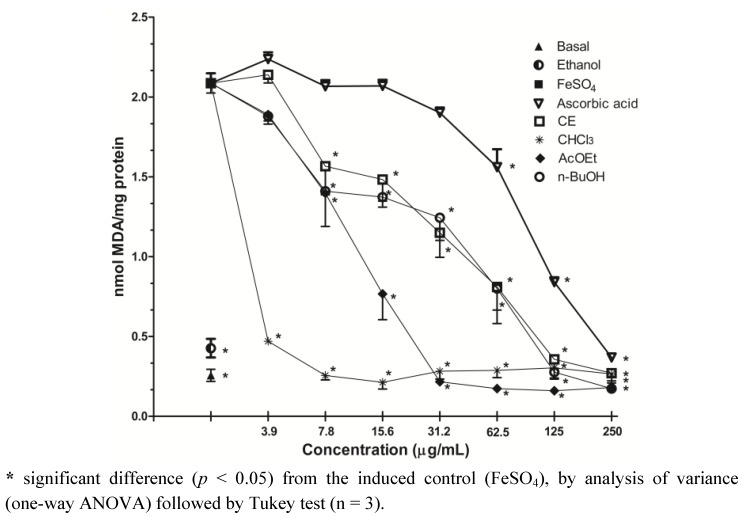
Effect of *S. guaraniticum* CE and fractions on TBARS production in supernatant of rat brain homogenate.

The antioxidant potential of the CE and fractions were evaluated through the TBARS assay based on the formation of malondialdehyde (MDA), a subproduct of lipid peroxidation. The lipid peroxidation was stimulated with FeSO_4_ addition to brain supernatant, and the extracts were able to significantly decrease of MDA formation at all concentrations tested when compared with the induced control, as can be observed in Figure 3. Similarly, all the extracts reduced MDA formation at the basal levels (non-induced group), especially the CHCl_3_ fraction, which showed a very low IC_50_ (Table 2). It is suggested that this effect is due to the large amounts of flavonoids, tannins and alkaloids found in this fraction, contributing to the antioxidant activity, and also due to the demonstrated ability to scavenge ROS. In our study, ascorbic acid showed an IC_50_ value of 117.81 ± 1.23 µg/mL, these results were in accordance with Liang *et al.*, [22] who found moderate inhibition of lipid peroxidation for ascorbic acid using a similar method. The vehicle (ethanol) in which all the extracts were dissolved had no effect when administered alone. Sini and Devi [23] observing the antioxidant activity of the chloroform extract of *Solanum trilobatum*, verified the capacity to inhibit lipid peroxidation in rat liver homogenate, and also the scavenging effect on superoxide and hydroxyl radicals whose results were higher than the standards butylated hydroxytoluene (BHT) and catechin.

### 2.5. Inhibition of Protein Oxidation-Carbonyl Content

**Figure 4:**
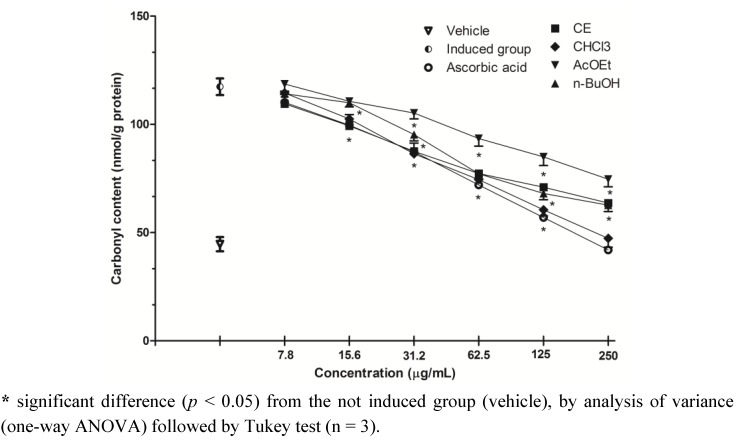
Effect of *S. guaraniticum* CE and fractions on protein carbonyl content in serum samples.

Protein carbonyl content is the most commonly used marker of protein oxidation, and its accumulation has been observed in several human diseases, including Alzheimer’s disease, diabetes, arthritis and others [24]. All the fractions were able to reduce protein carbonyl content in serum compared to the induced group (H_2_O_2_), in a dose-dependent way, but only the CHCl_3_ fraction, at the concentration of 250 µg/mL completely inhibited the oxidative damage caused (Figure 4). This fraction showed the second lowest IC_50_ for this assay (67.65 ± 0.82 µg/mL), slightly larger than the standard ascorbic acid (61.80 ± 0.54 µg/mL), however the difference between these two values was not significant (*p* < 0.05) (Table 2, Figure 4). Similarly to what was observed in the lipid peroxidation assay, this protective effect may be attributed to the phytochemical composition of the fraction and also the ability of scavenging ROS observed by DCFH-DA method, reducing the oxidative stress.

In our study, the CHCl_3_ fraction showed the best results by all antioxidant activity methods, except in the DPPH assay. The steric accessibility of DPPH radical is a major determinant of the reaction, since small molecules that have better access to the radical site have relatively higher antioxidant capacity. Moreover, many antioxidant compounds that react quickly with peroxyl radicals may react slowly or may even be inert in this assay. The inexistence of DPPH or similar radicals in biological systems is also a shortcoming. Despite these limitations, the DPPH radical is a simple, stable and useful spectrophotometric method with regard to screening measuring the antioxidant capacity of both pure compounds and complex samples [25,26].

### 2.6. HPLC/DAD Analysis

**Figure 5:**
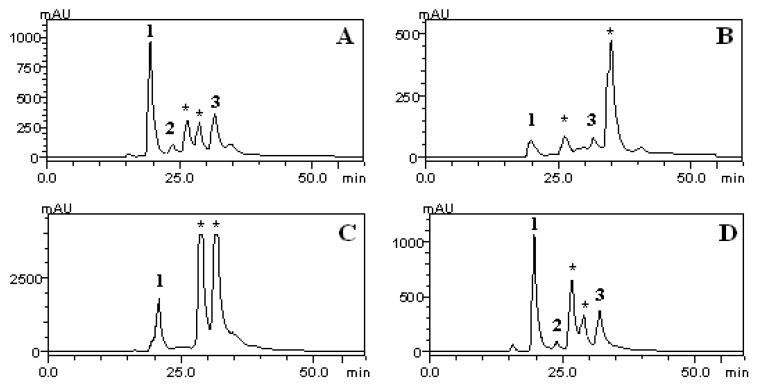
HPLC/DAD phenolics profile of CE (**A**), CHCl_3_ (**B**), AcOEt (**C**) and n-BuOH (**D**) fractions of *S. guaraniticum* leaves. (1) chlorogenic acid, (2) caffeic acid, (3) rosmarinic acid, (*) unknown peaks.

To verify the presence of phenolic compounds in the CE and fractions of *S. guaraniticum*, the samples were subjected to HPLC/DAD analysis. The phenolic acids such as chlorogenic, caffeic and rosmarinic were identified by comparison of their retention’s time and UV spectrum with those of the standards. The chromatograms and quantification of the compounds are shown in Figure 5 and Table 3, respectively.

**Table 3:** Quantification of polyphenols in CE and fractions of *S. guaraniticum* by HPLC/DAD.

Extract or fraction	CLA(mg/g)	CFA(mg/g)	RA(mg/g)
CE	11.15 ^b^ ± 0.14	1.80 ^a^ ± 0.15	51.92 ^a^ ± 5.58
CHCl_3_	3.23 ^c^ ± 1.04	-	2.01 ^b^ ± 0.1
AcOEt	21.55 ^a^ ± 0.73	-	-
n-BuOH	11.60 ^b^ ± 1.95	0.45 ^b^ ± 0.2	54.79 ^a^ ± 4.1

^a^^–c^ Means with the different letters in each column are significantly different (*p* < 0.05), by analysis of variance (n = 3). CLA = chlorogenic acid; CFA = caffeic acid; RA = rosmarinic acid.

Phenolic compounds may have different antioxidant capacities, depending on their structural conformation, number of hydroxyl groups and their distribution in the structure. In general, phenolic acids are considered efficient hydrogen donors due to their characteristic carboxylic acid group, which is easily ionized [27]. In this study, chlorogenic acid was identified in all extracts. However, the AcOEt fraction presented the highest amount of this phenolic acid (21.55 ± 0.73 mg/g), followed by CE (11.15 ± 0.14 mg/g), n-BuOH (11.60 ± 1.95 mg/g) and CHCl_3_ (3.23 ± 1.04 mg/g). These results follow the same trend of the antioxidant capacity by the DPPH assay, suggesting that this polyphenol was a significantly contributor to the low IC_50_ value presented by the AcOEt fraction in this method. 

Rosmarinic acid was present in high amounts in the CE and *n*-BuOH fractions. This phenolic acid is known to have many biological activities, including hepatoprotective activities in liver diseases [28] which could be related with the popular use of this plant. In fact, Sabir and Rocha [15] demonstrated the hepatoprotective activity of jurubeba in an experimental model using mice. Our findings corroborate the results obtained by those authors. Similarly, in a study of Lin *et al.* [29], flavonoids and phenolic acids in the ethanol extract from lemon balm leaves (*Melissa officinalis* L.) were evaluated, and rosmarinic acid, present in the amount of 83.33 ± 3.46 mg/g of freeze dried sample extract and 78.40 ± 4.13 mg/g of extract of hot air dried sample, was considered as the major component. In addition, caffeic acid has also been found in the CE and *n*-BuOH fractions, in small amounts, and may also have contributed to the activities shown.

Similar behaviors were found comparing the CE and *n*-BuOH fractions in relation to the content of polyphenols and flavonoids, antioxidant capacity by DPPH assay and lipid peroxidation. This fact can be explained, in part, by similarity of their chromatographic profiles and also by very similar amounts of the three phenolic acids as quantified by HPLC/DAD.

## 3. Experimental

### 3.1. Chemicals

All chemicals were of analytical grade. The solvents and reference compounds used for the extractions and analytical procedures such as chloroform, ethyl acetate, ethanol, *n*-butanol, gallic, chlorogenic and rosmarinic acids, and spectrophotometric grade methanol were purchased from Merck (Darmstadt, Germany). Folin-Ciocalteau reagent 2N, DPPH radical, bismuth nitrate, rutin, catechin, thiobarbituric acid and DCFH-DA were acquired from Sigma Chemical Co. (St. Louis, MO, USA). All others chemicals and reagents were purchased locally.

### 3.2. Plant Collection and Extractions

**Figure 6:**
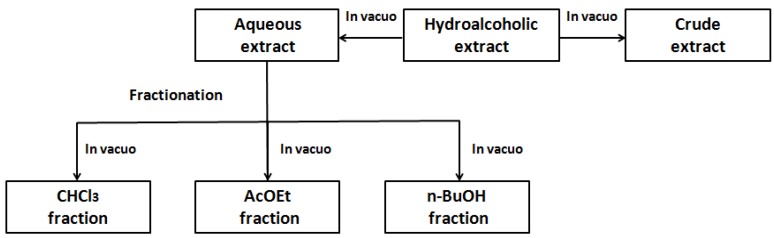
Method of fractionation of *S. guaraniticum* extract.

The leaves of *Solanum guaraniticum* were collected in December 2011, in the city of Guaporé (Rio Grande do Sul, Brazil). A voucher specimen is deposited in the herbarium of the Biology Department of Federal University of Santa Maria, and cataloged under the registration number SMDB 13158. Plant material (645.58 g) was dried in a stove at controlled temperature (38 °C), triturated and subjected to the maceration with 70% ethanol (4.3 L) for seven days, with daily agitation. The material was filtered and concentrated under reduced pressure, in order to obtain an aqueous extract. Part of this was taken to complete dryness, yielding the crude extract (CE), and another part was successively partitioned with chloroform, ethyl acetate and *n*-butanol, which also were dried to give each corresponding fraction. The fractionation method is illustrated below (Figure 6).

### 3.3. Phytochemical Analysis

#### 3.3.1. Total Polyphenols Content

The polyphenol content was evaluated by the colorimetric method described by Chandra and Mejia [30], using the Folin-Ciocalteau reagent. Samples were prepared at a concentration of 0.15 mg/mL. Absorbance was measured at 730 nm, in triplicate. Gallic acid was used to calculate the standard curve, and the results were expressed as mg of gallic acid equivalents per g of extract (mg/g GAE).

#### 3.3.2. Total Flavonoid Content

The total flavonoid content was determined according to the colorimetric method described by Woisky and Salatino [31], using a 2% aluminum chloride solution. Samples were prepared at a concentration of 1 mg/mL. Absorbance was measured at 420 nm, in triplicate. A standard curve of rutin was used for quantification, and the results were expressed as mg of rutin equivalents per g of extract (mg/g RE).

#### 3.3.3. Determination of Condensed Tannins

The determination of condensed tannins was performed according to the method described by Morrison *et al*. [32], using equal amounts of a 8% hydrochloric acid solution and a 1% vanillin solution. The samples were prepared at a concentration of 25 mg/mL, and the absorbance was measured at 500 nm. The test was performed in triplicate, for quantification was used a standard curve of catechin, and the results were expressed as mg of catechin equivalents per g of extract (mg/g CaE).

#### 3.3.4. Determination of Total Alkaloids

Total alkaloids were determined by reaction of precipitated with Dragendorff’s reagent, described by Sreevidya and Mehrotra [33]. The crude extract and fractions were prepared at a concentration of 50 mg/mL, absorbance was measured at 435 nm and the test was performed in triplicate. For calculating the total alkaloid content, a standard curve of bismuth nitrate was used, and the results were expressed as mg of total alkaloids per g of extract.

#### 3.3.5. HPLC/DAD Analysis

HPLC/DAD analysis was performed on a Shimadzu HPLC system (Kyoto, Japan), Prominence Auto-Sampler (SIL-20A), equipped with Shimadzu LC-20 AT reciprocating pumps connected to a DGU 20A5 degasser, CBM 20A integrator, UV–VIS detector DAD SPD-M20A and LC Solution 1.22 SP1 software. Reversed phase chromatographic analyses were carried out under gradient conditions using a C-18 column (250 mm × 4.6 mm) packed with 5 µm diameter particles. The phenolic acids analysis was carried out using a gradient system using Solvent A (water containing 2% acetic acid) and Solvent B (methanol), according to Evaristo and Leitão [34] with minor modifications. All solutions and samples were filtered through a 0.45 µm membrane filter (Millipore, Bedford, MA, USA), and the mobile phases were degassed by an ultrasonic bath prior to use. The flow rate was 0.8 mL/min and the injection volume was 40 µL. Identification of phenolics was performed by comparing retention times and the Diode-Array-UV spectra with those of standards. Stock solutions of chlorogenic, caffeic, and rosmarinic acids were prepared in the HPLC/DAD mobile phase at a concentration range of 0.00625–0.250 mg/mL. Samples of CE and fractions of *S. guaraniticum* were also dissolved in the mobile phase. Quantification was performed by integration of the peaks using the external standard method, and chromatographic operations were carried out in triplicate.

### 3.4. Animals

Male Wistar rats (3.0–3.5 months of age and weighing 270–320 g) were maintained groups of 3–4 rats per cage. They had continuous access to food and water in a room with controlled temperature (22 ± 3 °C) and on a 12 h light/dark cycle. The animals were maintained and used in accordance to the guidelines of the Brazilian Association for Laboratory Animal Science (COBEA). The rats were killed by decapitation and the brain tissue was rapidly dissected, weighed and immediately homogenized in Tris-HCl 10 mM, pH 7.5 (1/10, w/v). The homogenate was centrifuged for 10 min at 4,000 rpm and the supernatant was used for tests of scavenging of ROS by DCFH-DA method and inhibition of lipid peroxidation. 

### 3.5. Human Serum

For inhibition of protein oxidation (carbonyl content) assay, human blood samples were collected by venipuncture and separated by centrifugation to obtain the serum. The experiments were carried out according to the research ethics committee of the Federal University of Pampa (Rio Grande do Sul, Brazil), and approved under number 23,081.

### 3.6. Antioxidant activity Methods

#### 3.6.1. DPPH Radical Scavenging Capacity

The DPPH radical scavenging capacity of CE and fractions of *S. guaraniticum* were evaluated by the method described by Choi *et al.* [35]. Samples were diluted in ethanol at the following concentrations: 250, 125, 62.5, 31.25, 15.62 and 7.81 µg/mL. An aliquot of each dilution (2.5 mL) was added to a solution of 0.3 mM DPPH (1 mL), and the absorbance was measured at 518 nm against a blank after 30 min of reaction, in the dark and at room temperature. DPPH solution (1.0 mL) plus ethanol (2.5 mL) was used as a negative control, and ascorbic acid in the same concentrations was used as positive control. The DPPH scavenging ability was expressed as IC_50_ (the extract concentration required to inhibit 50% of the DPPH in the assay medium). The test was performed in triplicate, and the calculation of the antioxidant capacity followed the equation: 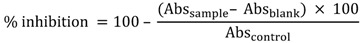
(1)
where Abs_sample_ is absorbance of each fraction; Abs_blank_ is absorbance of fractions without adding the DPPH; Abs_control_ is absorbance the solution of negative control.

#### 3.6.2. DCFH-DA Method

Intracellular formation of ROS was measured using 2′,7′-dichlorofluorescein diacetate (DCFH-DA) as the substrate, according to Myrhe *et al.* [36]. The supernatant of rat brain homogenate was incubating with different concentrations of CE and fractions of *S. guaraniticum*, at 37 °C. After 1 h, aliquots were removed and DCFH-DA (5 µM) was added to the medium and incubation continued for 1 h in the dark. Fluorescence was measured using 488 nm for excitation and 520 nm for emission. ROS levels (expressed as percentage value in relation to the control group) were calculated by interpolation in a standard curve of oxidized DCF (constructed in parallel), corrected by the content of protein [37]. Ethanol was used as negative control and ascorbic acid as positive control. The assay was performed in triplicate and data were expressed as mean ± S.E.M.

#### 3.6.3. Measurement of Inhibition of Lipid Peroxidation (TBARS Assay)

To evaluate the inhibition of lipid peroxidation, an aliquot of 100 µL of rat brain homogenate supernatant was incubated for 1 h at 37 °C with the pro-oxidant ferrous sulphate (10 µM) in the presence or absence of CE and fractions. The production of TBARS (thiobarbituric acid reactive substances) was determined by the colorimetric method according to Ohkawa *et al*. [38]. Quantification was expressed in nmol of malondialdehyde (MDA)/g of tissue. Ascorbic acid was used as positive control, and the extract concentration required to reduce the lipid peroxidation in 50% was expressed as IC_50_.

#### 3.6.4. Measurement of Inhibition of Protein Oxidation (Carbonyl assay)

The CE and fractions were diluted in PBS buffer in the desired concentrations. In a test tube, was placed 1 mL of serum and 1 mL of extract diluted in PBS buffer. After 30 min at 37 °C was added H_2_O_2_ 100 µm (final concentration). After 60 min at 37 °C, were performed dosages of protein carbonyl, according to Morabito* et al.* [39]. Briefly, 100 µL of serum in the absence or presence of CE and fractions in different dilutions was incubated with 100 µL of a 20 mM 2,4-dinitrophenylhydrazine (DNPH) solution for 60 min. The proteins were precipitated from the solution with the use of 20% trichloroacetate; the protein pellet was washed three times with ethanol and ethyl acetate and resuspended in 1 mL of 6 M guanidine at 37 °C for 15 min. The carbonyl content was determined from the absorbance at 366 nm (molar absorption coefficient, 22.000 M^−1^/cm). The determination of total protein in serum was conducted using a commercial Labtest^®^ kit as recommended by the manufacturer. All tests were performed in triplicate, and the carbonyl content was expressed as nmol/g protein. Ascorbic acid was used as positive control. The extract concentration required to reduce the damage to proteins in 50% was expressed as IC_50_.

### 3.7. Statistical Analysis

For phytochemical composition, antioxidant capacity by DPPH assay and HPLC/DAD analysis, was used a calibration curve, and the experimental values were statistically analyzed by analysis of variance. Data were expressed as mean ± S.D. (n = 3). For the data analysis of inhibition of lipid peroxidation, protein carbonyl content and scavenging of ROS by DCFH-DA method, was used analysis of variance (one-way ANOVA) followed by Tukey test, and *p* < 0.05 were considered significant. Data were expressed as mean ± S.E.M.

## 4. Conclusions

This study showed the antioxidant activity of the leaves of *S. guaraniticum* and contributed to reveal some phytochemical characteristics of this species. The AcOEt fraction showed the better antioxidant activity by the DPPH assay, which can be attributed to its high content of total polyphenols. In addition, the CHCl_3_ fraction that presented high values of flavonoids, tannins and alkaloids, was also shown to be able to remove reactive species by the DCFH-DA method, reducing oxidative stress, lipid peroxidation and damage to proteins. Chlorogenic, caffeic, and rosmarinic acids were identified and quantified for the first time in this species by HPLC/DAD and may be involved in the activities shown. Additionally, the presence of high contents of rosmarinic acid in the crude extract and *n*-butanol fraction may support, at least in part, the popular use of the plant to treat liver diseases. Taken together, our results indicate that this plant has antioxidant potential and can be a promising source of natural antioxidants. 
